# Pathological findings of slaughtered camels’ *(Camelus dromedaris) *kidneys in Najaf-Abad, Iran

**Published:** 2014

**Authors:** Gholam Ali Kojouri, Hossein Nourani, Sirous Sadeghian, Hadi Imani, Abbas Raisi

**Affiliations:** 1 *Department of Clinical Sciences, School of Veterinary Medicine, Shahrekord University, Shahrekord, Iran;*; 2 * Department of Pathobiology, Faculty of Veterinary Medicine, Ferdowsi University of Mashhad, Mashhad, Iran; *; 3 *Department of Internal Medicine, Faculty of Veterinary Medicine, Tehran University, Tehran, Iran;*; 4 * Department of Clinical Sciences, Faculty of Veterinary Medicine, Shahid Chamran University of Ahvaz, Ahvaz, Iran; *; 5 *Department of Clinical Sciences, Faculty of Veterinary Medicine, Lorestan University, Khorram Abad, Iran.*

**Keywords:** Camel, Histopathology, Kidney

## Abstract

The kidney of camel is known to play a vital role in water conservation through the production of highly concentrated urine that may predispose animal to varieties of renal dysfunction. In camels renal disorders have received lesser attention in comparison with other animals, thus there is shortage of information in this area. The present study was conducted on 100 slaughtered camels *(Camelus dromedaris)* (200 kidneys) in Najaf-Abad district (Iran) to evaluate the frequency and types of renal disorders. Results demonstrated varieties of gross abnormalities in 14.00% of kidneys that out of them, 9.00% were confirmed by microscopic examination. Renal capsular pigmentation, medullary hyperemia, subcapsular calcification, cortical and medullar discoloration, hemorrhage in renal pelvis, nephrolithiasis and hydatidosis were recorded in 3, 6, 5, 6, 3, 2 and 3 cases, respectively. In addition, capsular melanosis, acute tubular necrosis, chronic interstitial nephritis, caseous necrosis, calcification, medullary hyperemia, and hydatid cyst were confirmed by histopathological examination in 3, 5, 1, 3, 2, 2, and 2 cases, respectively. Our findings indicate the presence of many types of renal disorders which may relate to dehydration, bacteremia or nephrotoxicosis. In addition capsular melanosis in male camel was recorded for the first time and its etiology remains to be addressed.

## Introduction

Kidneys of camel are bean shaped with a very strong, thick and completely adhesive capsule.^1^ Generally, kidneys excrete the end-products of tissue metabolism and maintain fluid, electrolyte and acid-base balance via varying the volume of water and concentration of solutes in urine. Abdalla and Abdalla stated that camel’s kidney possesses anatomical requisites for production of hypertonic urine. The renal cortex in camel occupies about 50% of the kidney’s volume, and the ratio of thickness of the medulla to cortex has been evaluated about 4:1. The relative thickness of the medulla is about 7.89 cm. This parameter is an indicator of the length of Henle and vasa recta loops, and according to the countercurrent theory, is consequently an indicator of the kidney ability for urine concentrating.^2^

According to previous histopathological study on camel kidney, renal corpuscles and glomeruli are larger than those of other domestic animals.^3^ Reportedly, dehydrated camels had 73.00% decreases in tubular reabsorption of sodium leading to an increase of urinary sodium excretion by 42.00%.^4^ Many investigators explained the different causes of renal insufficiency in domestic animals and divided it into prerenal, renal, and postrenal groups.^5^^,^^6^ Nephritis and glomerulonephritis are relatively rarely reported in camelids.^1^ Conversely, the prevalence of these disorders was reported in 34.18 and 3.80 percent of slaughtered cattles, respectively.^5^ These differences may play an important role in diagnosis, treatment, and prognosis of camelids cases showing urinary system involvements. For these reasons, the present study was designed to determine the prevalence of renal disorders in one-humped slaughtered camel. 

## Materials and Methods 

The present study was conducted in Najaf-Abad slaughter house (Isfahan province, Iran) from May 2009 to January 2010 to estimate the frequencies and relative frequencies of acquired disorders of camel kidney. Eighteen female and 82 male camels *(Camelus dromedaris)*, aged between eight to ten years (except for two male with ages 3 and 30 years), were inspected. During antemortem examinations, each camel was given an identification number and age, sex, and origin of animals were recorded. The ages of the animals were recorded according to dental formula.^1^ Precise inspections were carried out on all organs of the animals in abattoir, including lung, liver, spleen, kidney, heart, and the muscles. Each organ was accessed macroscopically either by visual inspection or palpation, and one or more incisions were made in order to detect the disorder if required. The kidneys were inspected carefully and their position and size of probable macro-scopic lesions were recorded. In second step, longitudinal sections were made on the kidneys for identification of lesions in subcapsular, cortical, and medullary sections. Renal crest, medullary pyramids, and renal pelvis were carefully observed for determination of hemorrhage and calculus presence. For the histopathological investigations, the tissue specimens were taken from different regions of the kidneys including gross pathologic lesion, fixed in buffered formalin solution and processed through routine paraffin embedding technique then cut at 5 μm and stained with hematoxylin and eosin (H & E) under light microscopy.

Chi-square test was used for determination of the relation between sex, age and the frequency of disorders at the level of *p*<0.05.

## Results

Results indicated that the prevalence of renal diseases in slaughtered camel kidneys were 14.00% and based on our findings, 28.04, and 27.77 percent of male and female kidneys had macroscopic lesions, respectively (Table 1). Further, the relative frequency of lesions located on dorsal surface (83.34%) of kidneys was significantly higher than those located on ventral (16.66%) surface (*p *< 0.05). 

**Table 1:** Frequency of macroscopic acquired disorders of camel kidneys based on sex and age

**Groups**	**Number of cases**		**Abnormalities**			
	**Male**	**Female**	**Male**		**Female**	
			**Right**	**Left**	**Right**	**Left**
**< 8 years**	1	-	1	-	-	-
**8-10 years**	80	18	12	8	2	3
**> 10 years**	1	-	1	1	-	-
**Total**	82	18	14	9	2	3
**Percent**			**28.04%**		**27.77%**	

**Fig. 1:**
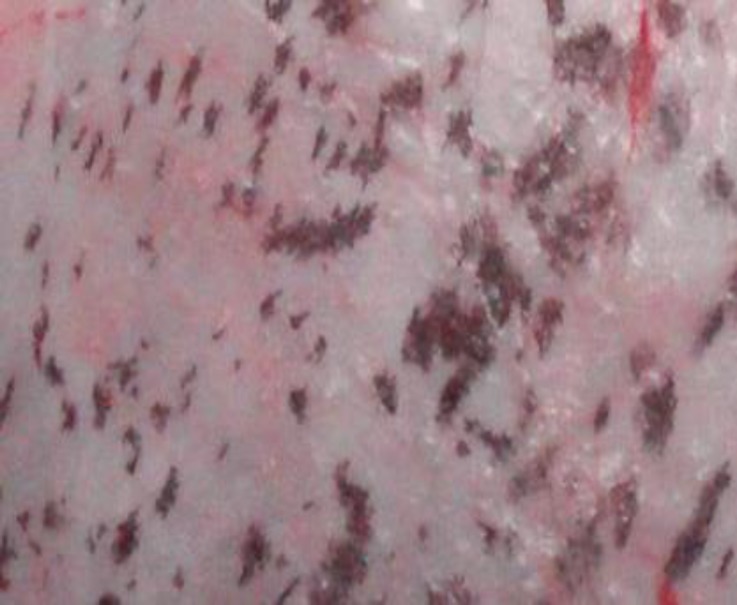
Renal capsular pigmentation in one-hump male camel

**Fig. 2:**
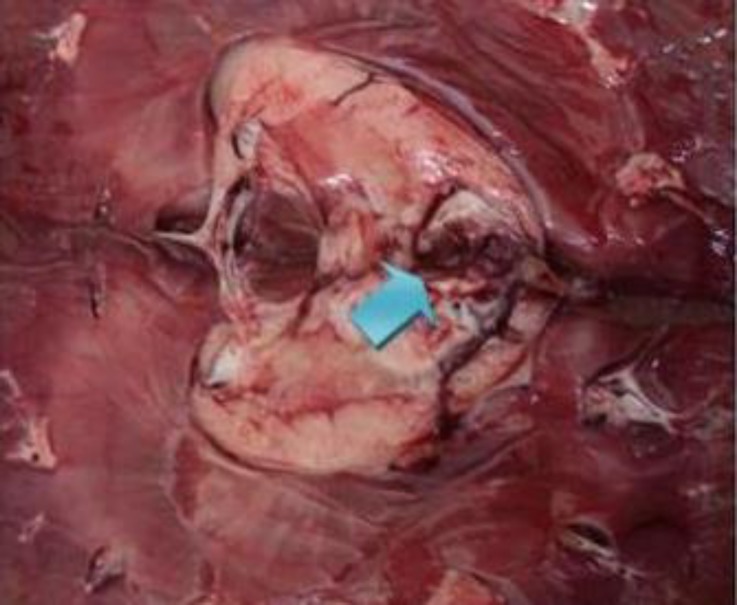
Hemorrhage and hyperemia (arrow) in the renal pelvis

**Fig. 3:**
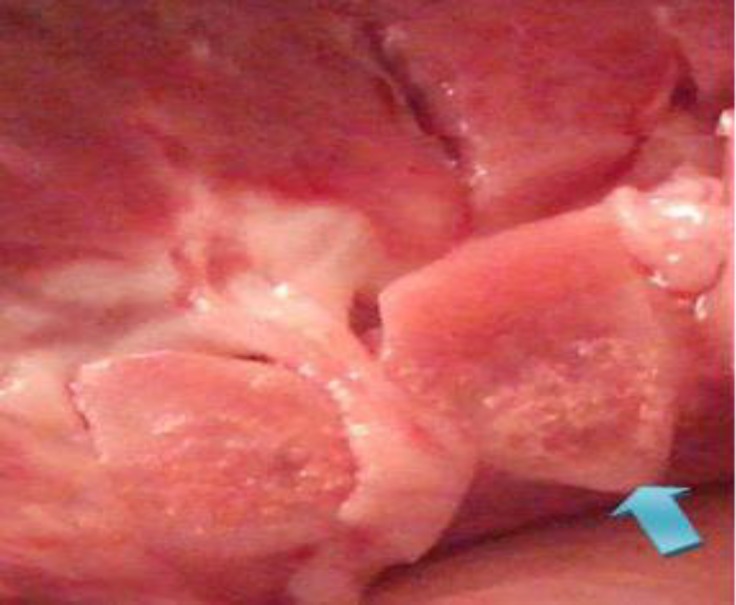
Inner medulla and papillary necrosis

**Fig. 4:**
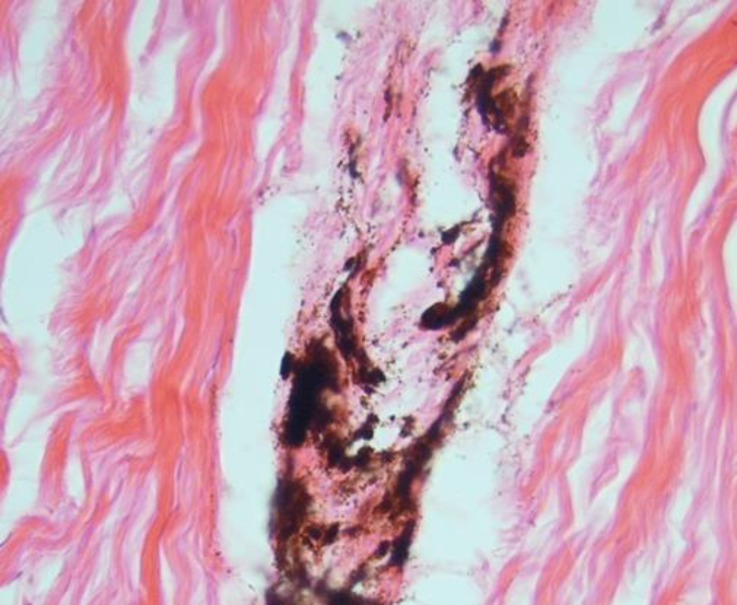
Renal capsular melanosis, (H&E, 528×).

**Fig. 5:**
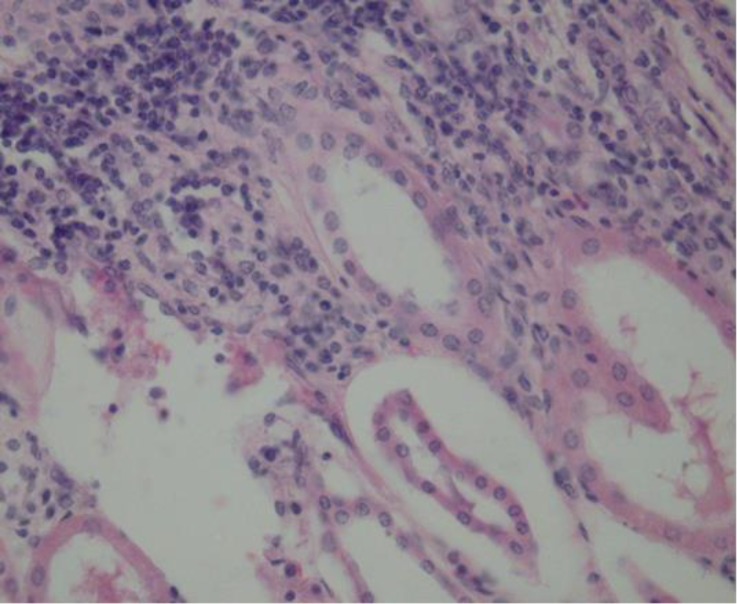
Chronic interstitial nephritis, (H&E, 528×).

As shown in Table 2, renal capsular pigmentation (Fig. 1), medullar hyperemia, cortical and medullar discoloration, renal pelvis hyperemia and hemorrhage (Fig. 2), nephrolithiasis, inner medulla and papillary necrosis (Fig. 3), sub capsular calcification, and hydatidosis were recorded during gross examination, of which renal capsular pigmentation was only observed in male camels. The histopathological findings confirmed capsular melanosis (Fig. 4), acute tubular necrosis, caseous necrosis, chronic interstitial nephritis (Fig. 5) calcification, medullary hyperemia, and hydatid cyst, of which acute tubular necrosis was recorded in higher level than the other lesions (Table 3).

**Table 2:** Frequency of macroscopic acquired disorders of camel kidneys based on sex and age

**Disorder**	**Frequency**	**Relative frequency**
Capsular pigmentation	3	10.72%
Medullar hyperemia	5	17.85%
Subcapsular calcification	4	14.28%
Cortical & medullar discoloration	5	17.85%
Pelvis hyperemia/hemorrhage	6	21.43%
Nephrolithiasis	2	7.15%
Hydatidosis	3	10.72%
**Total**	**28**	**100%**

**Table 3:** Frequency and relative frequency of microscopic abnormalities of camel kidneys

**Disorder**	**Frequency**	**Relative frequency**
Capsular melanosis	3	16.67%
Acute tubular necrosis	5	27.78%
Chronic interstitial nephritis	1	5.55%
Caseous necrosis	3	16.67%
Calcification	2	11.11%
Medullary hyperemia	2	11.11%
Hydatid cyst	2	11.11%
**Total**	**18**	**100%**

## Discussion

Renal function depends on the number and function of the individual nephrons. Insufficiency can occur from abnormalities in the rate of renal blood flow, the glomerular filtration rate and the efficiency of tubular re-absorption. Of these three abnormalities, the latter two are intrinsic functions of the kidney, whereas the first depends largely on vasomotor control which is markedly affected by circulatory emergencies such as shock, dehydration, and hemorrhage.^6^

The kidney of camel is known to play a vital role in water conservation through the production of highly concentrated urine that may predispose animal to many kinds of renal dysfunction. There are few surveys on kidney disease in slaughtered camel. Renal disorders have received much less attention in camel than in other animals and there is still a lack of knowledge in this field. However, most renal lesions are subclinical and they might have remarkably higher frequencies than expected. Such lesions could result in the poor production of the involved animals. In this investigation the presence of 28 kinds of gross abnormalities were revealed and 18 cases were confirmed by histopathological examination. According to the report in the cattle 4.20% of rejected carcasses in the abattoir is due to focal interstitial nephritis.^7^ In another slaughterhouse survey,^8^ gross signs of pyelonephritis were found in twenty one rejected kidneys in Pennsylvania. 

Among farm animals, bladder and urethral diseases are more common and more important than diseases of the kidneys.^6^ Occasionally, renal insufficiency develops as a sequel to diseases such as pyelonephritis, embolic nephritis, amyloidosis, glomerulonephritis, nephrosis and many other diseases. Many investigators explained the different causes of renal insufficiency in domestic animals and categorized it into prerenal, renal, and postrenal groups.^6^ Our findings confirmed the presence of acute tubular necrosis (2.50%), chronic interstitial nephritis (0.50%), caseous necrosis (1.50%), calcification (1.00%), medullary hyperemia (1.00%) and hydatid cyst (1.00%) in 100 paired camel kidneys. The present data can show the importance of renal diseases in camel that is known to play a vital role in water conservation through the production of highly concentrated urine.^9^ Like other domestic animals, nephrosis may occur as a result of high doses administration of aminoglycosides antibiotics (such as gentamycin) and non-steroidal anti-inflammatory drugs like flunixin meglumine and phenylbutazone, especially in dehydrated camel. Dilation of renal pelvis due to obstruction of ureters, bladder, or urethra may cause hydronephrosis and destruct the kidney tissues.^6^ Zguigal and Ouhsine revealed that the presence of recesses in the renal pelvis of camel kidney is the most important anatomical characteristic for movements of solute from pelvic urine into the medullar tissue, and thus, assist in building up urea and the osmotic concentration in papillary tissues.^10^

The occurrence of urine recycling, water re-absorption and the presence of concentrated urine may highly expose camel kidneys to the risk factors. Uzal *et al.*, suggested that neither *Leptospira spp.*, nor active infections due to other bacteria had a role in formation of bovine focal chronic interstitial nephritis. ^11^ Conversely, the role of bacteremia in developing the cases of septicemic colibacillosis and infections by *Salmonella spp*. or *Brucella spp.* was reported.^12^^,^^13^ Some investigators explained the types of uroliths in dromedaries that some of them had a large proportion of calcium,^14^^,^^15^ necrotic inflammatory cells without mineral contents,^16^ or silica.^17^^,^^18^

Our findings indicated the presence of many types of renal disorders not limited to a specific region and as shown in Figure 1, renal capsular pigmentation was reported for the first time. It needs further investigations to find the main causes in male one-hump camel.

As discussed above, many conditions can result in renal insufficiency and it is difficult to determine these causes. Thus, abattoir surveys like the present study may play an important role in such areas to find the high frequency lesions and diminishing animal exposure to probable etiological agents. 

Our findings indicated the presence of many types of renal disorders which may be related to dehydration, bacteremia or nephrotoxicosis.
